# Integrated approach for estimating climate change impacts on CO_2_ sink capacity of inland waterbodies using hydrodynamic modelling and GIS analysis

**DOI:** 10.1038/s41598-024-81707-1

**Published:** 2025-01-04

**Authors:** Hanady H. Khalil, Mohamed A. Abdrabo, Mahmoud A. Hassaan, Mohamed M. Elshemy

**Affiliations:** 1 Institute of Graduate Studies and Research, Environmental Studies, Alexandria Research Center for Adaptation to Climate Change (ARCA), Alexandria University, Alexandria, Egypt; 2 Department of Irrigation and Hydraulics Engineering, Faculty of Engineering, Tanta University, Tanta, Egypt; 3 Department of Civil Engineering, Faculty of Engineering, Al-Baha University, Al-Baha, Saudi Arabia

**Keywords:** CO_2_ sink capacity, Climate change, Climate change scenarios, Hydrodynamic model, Delft-3D, Inland waterbodies, GIS, Climate sciences, Environmental sciences, Hydrology

## Abstract

As one of their key regulatory ecosystem functions, inland lakes serve as CO_2_ sinks. The CO_2_ sink capacity of inland lakes depends on their water temperature and salinity as well as their water volume which are all highly sensitive to climate conditions. This paper aims to quantitatively estimate the change in the CO_2_ sink capacity of Wadi El-Rayan Lakes under climate change scenarios. For this purpose, an integrated approach combining CO_2_ solubility modelling, hydrodynamic simulations (Delft3D-FLOW) and GIS analysis was employed. According to the developed approach, CO_2_ solubility under variable temperature and salinity is mathematically modelled and this model is further used with the developed hydrodynamic model data for Wadi El-Rayan Lakes (temperature, salinity and water depth) to estimate their CO_2_ sink capacities. CO_2_ sink capacity is estimated for 2014 and 2050 under two Representative Concentration Pathways (RCPs) 2.6 and 8.5. Afterwards, the alteration in CO_2_ sink capacities due to climate change is determined using the modified hydrodynamic model. The results revealed that by 2050, the lakes would lose about 23–25% of their capacities compared to that of 2014 according to RCP 2.6 and 8.5, respectively.

## Introduction

Climate change adaptation strategies aim to stabilize the atmospheric concentrations of Greenhouse Gases (GHGs) through reducing emission or enhancing sinks of GHGs. Carbon dioxide (CO_2_) is the main GHG that is responsible for global warming which leads to climate change. Sinks of CO_2_ absorb it from the atmosphere and hence reduce its concentration. Generally, waterbodies, vegetation and soil act as sinks for CO_2_ as one of their ecosystem regulatory functions. Waterbodies dissolve CO_2_ from atmosphere to form carbonic acid (H_2_CO_3_) (Eq. 1), which can be disassociated again into CO_2_ and water molecules (H_2_O), or forming bicarbonates (HCO_3_^-^) (Eq. 2) and carbonates (CO_3_^–2^) (Eq. 3).These reactions depend on temperature, pH and/or atmospheric concentration of CO_2_^1–3^. Also, CO_2_ is consumed by phytoplankton in waterbodies in the photosynthesis process to generate oxygen.

1 $${CO}_{2}+{H}_{2}O\leftrightarrow {H}_{2}{CO}_{3}$$

2 $${H}_{2}{CO}_{3}\leftrightarrow {H}^{+}+{HCO}_{3}^{-}$$

3 $${H}_{2}{CO}_{3}\leftrightarrow {2H}^{+}+{CO}_{3}^{-2}$$

Global warming and associated increase in waterbody’s temperature usually accelerate evaporation and reduce the CO_2_ solubility (Table 1). Evaporation, in turn, affects water volume in which CO_2_ would dissolve and consequently would affect the CO_2_ concentration in the atmosphere. Therefore, the relationship between waterbodies and climate change is mutual and dynamic. In this context, preserving waterbodies by successful management would help in climate change mitigation by enhancing the CO_2_ sink.

**Table 1:** Solubility of carbon dioxide (mg/L) in water at different temperatures and salinities exposed to moist air containing 0.04% carbon dioxide at a total air pressure of 760 mmHg^16^.

Temperature	Salinity ppt								
(°C)	0	5	10	15	20	25	30	35	40
0	1.34	1.31	1.28	1.24	1.21	1.18	1.15	1.12	1.09
5	1.1	1.08	1.06	1.03	1.01	0.98	0.96	0.93	0.89
10	0.93	0.91	0.87	0.85	0.83	0.81	0.79	0.77	0.75
15	0.78	0.77	0.75	0.73	0.7	0.68	0.66	0.65	0.64
20	0.67	0.65	0.63	0.62	0.61	0.6	0.58	0.57	0.56
25	0.57	0.56	0.54	0.53	0.52	0.51	0.5	0.49	0.48
30	0.5	0.49	0.48	0.47	0.46	0.45	0.44	0.43	0.42
35	0.44	0.43	0.42	0.41	0.4	0.39	0.39	0.38	0.37
40	0.39	0.38	0.37	0.36	0.36	0.35	0.35	0.34	0.33

To predict climate change impacts on the CO_2_ sink, in a given waterbody, there would be a need for modelling and simulating the hydrodynamics of this waterbody. Such a simulation can provide insight into how the CO_2_ sink would be affected by climate change which in turn would give ideas about how much effort is to be given to mitigate and/or adapt to the change. Knowing the current and/or future sink capacities depends, to great extent, on understanding and then modelling the complex relationship between the waterbody’s temperature and salinity in one hand and the CO_2_ solubility in the other hand. Successful modelling of such complex relationship leads to better estimation of the waterbody potentials to dissolve CO_2_ from the atmosphere. 

Climate change and CO₂ sinks in waterbodies are closely interconnected due to the crucial role that waterbodies play in carbon sequestration as they are significant carbon sinks, absorbing roughly a quarter of the CO₂ emitted by human activities each year. This absorption helps moderate global temperatures by reducing the amount of CO₂ in the atmosphere, which in turn limits the greenhouse effect and slows climate change^4^.

Generally, the CO_2_ sink, as an ecosystem function of inland waterbodies, was repeatedly considered by a number of previous studies. For example, Raymond et al., (2013) provided an estimate for regional variations in global inland water surface area, dissolved CO_2_ and Carbon dioxide (CO_2_) transfer from inland waters to the atmosphere^5^. Other studies explained the biogeochemical and hydrological mechanisms driving CO_2_ concentrations in inland waters^6,7^. Usually, mega development projects and associated environmental and socioeconomic changes are expected to have significant impacts on ecosystem function as CO_2_ sink. In this respect, seasonal and annual fluxes of CO_2_ emissions from Chinese inland waterbodies was quantified and their changes during the period 1980s–2010s was evaluated^8,9^. Due to the essential role of GHGs emission of inland waterbodies in understanding their role as a CO_2_ sink, several studies attempted to estimate such emissions in different parts of the world^10,11^.

It was noted that different previous studies emphasized the significant impacts of climate change on the ecosystem functions of inland waterbodies as CO_2_ sink. Several literatures employed sampling, physiochemical analysis to lake water and/or sediment and statistical analysis to study the lakes’ CO_2_ sinks^12–15^. Conversely, integrating modelling CO_2_ solubility in water, hydrodynamic modelling and GIS analysis to study the CO_2_ sink capacity of a waterbody is still a little explored area of research. Moreover, limited number of previous studies, to our knowledge, provided estimates for the change in the waterbodies CO_2_ sink capacity in the future under different scenarios of climate change in general and specially for Egyptian lakes. Therefore, for the Middle East inland waters, this study can be considered as the first research work investigating this topic.

The aim of this work is to propose an integrated approach to quantitatively estimate the change in a waterbody CO_2_ sink capacity due to climate change by using hydrodynamic modelling and GIS analysis. Such an integrated approach can support climate change mitigation strategies as it provides an insight into the waterbody CO_2_ sink.

## Material and methods

### Study area

**Fig. 1:**
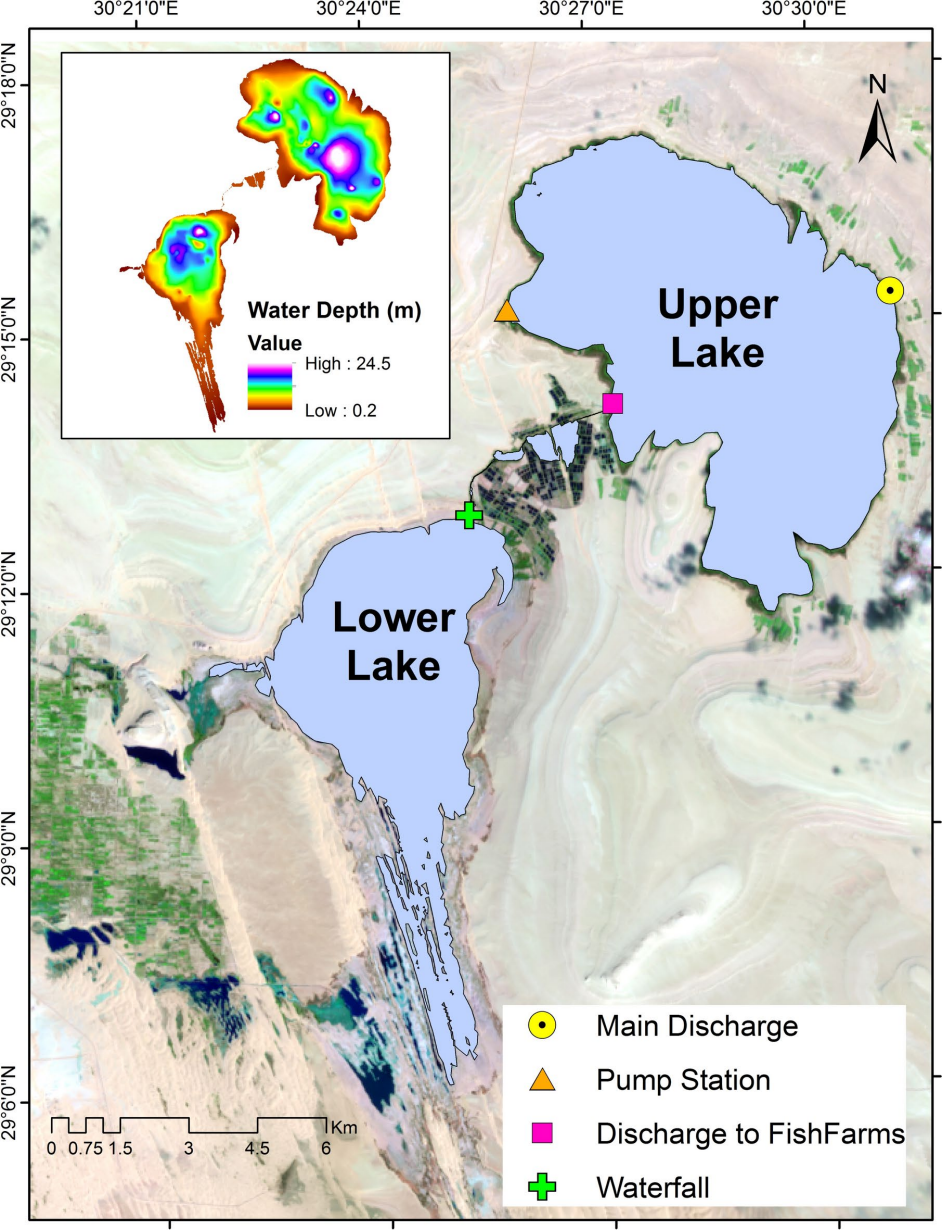
Wadi El-Rayan Lakes, geographic location, inlets and intakes and water depth.

Wadi El-Rayan Lakes are two man-made lakes in the hyper-arid Western Desert of Egypt near Fayoum (Fig. 1)^17–19^. Their origin is traced back to 1973 when agricultural wastewater was diverted to Wadi El-Rayan depression forming the Upper Lake first and in 1980 the surplus water that exceeds the Upper Lake’s holding capacity fell through a waterfall to form the Lower Lake^20–22^. The two lakes are at different levels in relation to the mean sea level^17–19,23,24^: the Upper Lake is at − 10.00 m AMSL, while the Lower Lake is at − 32.00 m AMSL^25,26^. The Upper Lake obtains its water from a diversion of a main drain called “Wadi Drain”^27^ and gives water to nearby reclaimed lands and fish farms^18,28^. The Lower Lake is a semi-closed basin that receives its water from the Upper Lake’s surplus water and some minor drains from nearby agricultural lands and the main water loss is by evaporation^29^. The inlet and the intake of the waterbody (Fig. 1) can be pointed out as:

- The main discharge to the waterbody which comes from the Wadi Drain through a 9 km canal then an 8 km tunnel and dispenses its water in the north-east of the Upper Lake^27^.
- The pump station which is located in the west of the Upper Lake and it is responsible for the irrigation of the reclaimed lands in the west.
- The discharge to the fish farms that depends on the Upper Lake’s water^30^.

Wadi El-Rayan Lakes are located in a hyper-arid area that is characterized by hot and dry climate with high evaporation rates exceeding 4.65 mm/day on average^31^, and low precipitation^17,23,32^. The prevailing wind is Northern, varying from North-Western to North-Eastern^33^ resulting in the formation of extensive sand dunes^34,35^.

The two lakes have different salinity levels ranging between 2‰ in the Upper Lake and 13‰ in the Lower Lake^29,36–39^. This remarkable difference in salinities may imply that they have two different ecosystems.

### Data and methodology

**Fig. 2:**
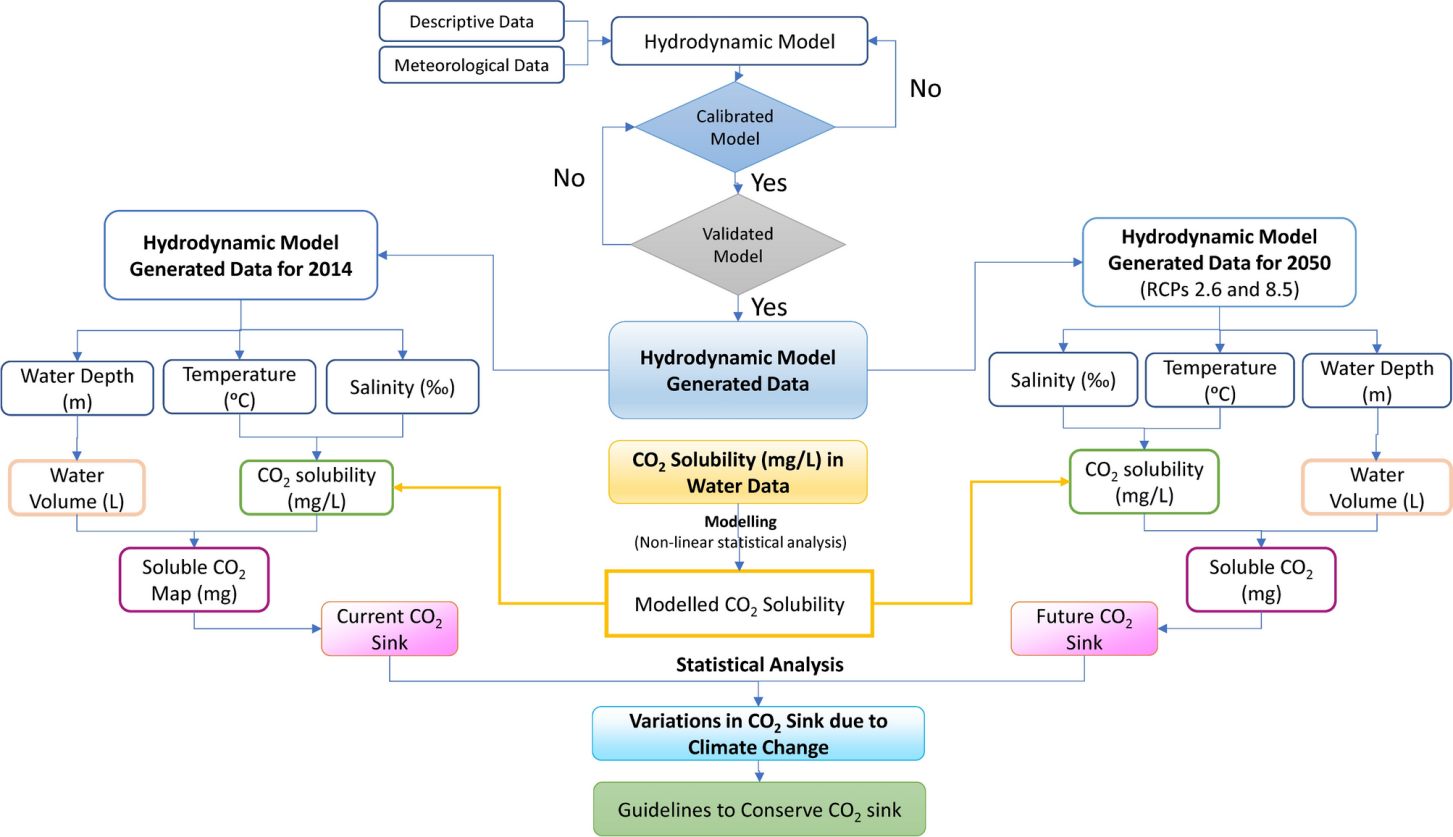
Methodology flowchart.

To estimate the impacts of climate change on the CO_2_ sink capacity under different scenarios, a methodology of four main steps was applied including simulating the CO_2_ solubility (mg/L) as a function of temperature and salinity, developing a hydrodynamic model for Wadi El-Rayan Lakes, predicting key physical and chemical parameters of Wadi El-Rayan Lakes under climate change scenarios, and finally estimating the change in Wadi El-Rayan CO_2_ sink capacity (Fig. 2).

Miscellaneous data are used in this procedure. Some of them are generated from tabulated data, interpolated from given points or contour maps (Table 2).

**Table 2:** Data input, their source and their output.

Input	Source	Output
Vector layer for Wadi El-Rayan Lakes	Generated by the author using remote sensing Landsat image for the year 2015 and GIS	Boundary file for Delft3d-FLOW
Tabulated data for temperature and salinity in 7 monitoring stations with given coordinates	^36,37^	Vector layer for monitoring stations Temperature distribution map for Wadi El-Rayan Lakes Salinity distribution map for Wadi El-Rayan Lakes
Contour map for the depth of Wadi El-Rayan Lakes	^38^	Raster layer for the depth of Wadi El-Rayan Lakes
Flow data (inflow and intake)	^30^	Discharge files for Wadi El-Rayan Lakes
Water level charts for Wadi El-Rayan Lakes	^25^	Calibration and validation of the hydrodynamic model of Wadi El-Rayan Lakes
Meteorological data 2013, 2014, 2015 and 2050 (RCPs 2.6 and 8.5) Air temperature (°C or °K) Relative humidity (%) Cloudiness (%) Solar radiation (W/m^2^) Wind speed (m/s) Wind direction Wind stress (Pa) Surface downwelling clear sky shortwave radiation (W/m^2^)	^40–42^	Heat flux model file
Boundary file Vector layer for monitoring stations Temperature distribution map for Wadi El-Rayan Lakes Salinity distribution map for Wadi El-Rayan Lakes Raster layer for the depth of Wadi El-Rayan Lakes Discharge files for Wadi El-Rayan Lakes Heat flux model file	This work	Hydrodynamic model set-up, calibration and validation
Tabulated data about the solubility of carbon dioxide (mg/L) in water at different temperatures and salinities exposed to moist air containing 0.04% carbon dioxide at a total air pressure of 760 mmHg	^16^	Model for CO_2_ solubility in water
Temperature distribution maps of Wadi El-Rayan Lakes for the years 2014 and 2050 (according to RCPs 2.6 and 8.5) Salinity distribution maps of Wadi El-Rayan Lakes for the years 2014 and 2050 (according to RCPs 2.6 and 8.5) Model for CO_2_ solubility in water	This work	Surface CO_2_ solubility maps for the years 2014 and 2050 (according to RCPs 2.6 and 8.5)
Water depth maps of Wadi El-Rayan Lakes for the years 2014 and 2050 (according to RCPs 2.6 and 8.5)	Output results of the hydrodynamic model in this work	Water volume (L) maps of Wadi El-Rayan Lakes for the years 2014 and 2050 (according to RCPs 2.6 and 8.5)
Surface CO_2_ solubility maps of Wadi El-Rayan Lakes for the years 2014 and 2050 (according to RCPs 2.6 and 8.5) Water volume maps of Wadi El-Rayan Lakes for the years 2014 and 2050 (according to RCPs 2.6 and 8.5)	Output of this work	Soluble CO_2_ in Wadi El-Rayan Lakes maps for the years 2014 and 2050 (according to RCPs 2.6 and 8.5)

### Modelling the CO_2_ solubility at different temperatures and salinities

At constant CO_2_ partial pressure above the waterbody, the CO_2_ solubility depends on both temperature and salinity. The lakes’ water temperature and salinity are changeable through different times of the year. These changing parameters of temperature and salinity control the amount of soluble CO_2_ in water. As both water temperature and salinity increase, the solubility of CO_2_ decreases^16^. Data given in Table 1 is used for mathematical analysis to model the CO_2_ solubility (mg/L) in water at different temperatures and salinities assuming two conditions:

1. The waterbody is exposed to moist air containing 0.04% CO_2._
2. The total air pressure is 760 mmHg (1 atm).

A non-linear mathematical analysis is used to related CO_2_ solubility (mg/L) to both temperature (°C) and salinity (‰) using OriginLab software (OriginPro 2016, b9.3.226)^43^ which is a high-performance statistical analysis software. The fit equation is used later to quantitatively estimate the potential soluble amount of CO_2_ in a known volume of water (lake’s water).

### Developing a hydrodynamic model for Wadi El-Rayan Lakes

To simulate hydrodynamic flow of Wadi El-Rayan Lakes, Delft3D-FLOW model was selected because it is an open-source, relatively easy to use, well-documented and technically supported modelling software. Besides it is used extensively and successfully to simulate lake hydrodynamics in different parts of the world^44–52^. A representative hydrodynamic model goes through three steps: model set-up, calibration and validation. Model set-up requires descriptive data about Wadi El-Rayan Lakes in addition to meteorological data. Descriptive data includes the geometrical boundaries of Wadi El-Rayan Lakes, the bathymetry, water level, temperature and salinity, bottom roughness and water viscosity besides the quantities of water inflow and intake. Meteorological data includes air temperature, humidity, cloud coverage, solar radiation, wind speed and direction. Model calibration includes the comparison of the model outputs with ground truth data such as water level, for example, and the adjustment of the model’s different parameters such as roughness and viscosity, for example, in order to yield as close values to ground truth as possible. In some rare cases, adjustments to water flow can be done to compensate for a source of water gain or loss that is not included in the model set-up process. Model validation is a performance test made for the calibrated model using a different set of data rather than those used in the model set-up and calibration and compare the model output to ground truth data using error matrices. If the error in the validation process is acceptable, the model is then representative to the waterbody under investigation.

#### Model set-up

The main steps to set-up the model are as follows:

- Define model dimensionality according to the purpose of the study and the data availability.
- Delineate the waterbody physical domain and construct the suitable grid representing it.
- Specify the time frame for the simulation process.
- Determine driving forces working on the waterbody and provide time series data covering the selected time frame.
- Identify the discharges to and from the waterbody and provide time series data for their quantities.
- Indicate the initial conditions at the beginning of the simulation.

For model set-up, two-dimensional (2D) model is used to represent Wadi El-Rayan Lakes. 2D model is simple enough to attain the goal of this work and complies with available data. Keeping the model as simple as needed saves the time and effort of the processing and requires less data storage capabilities. A mesh of 75 × 107 is created to represent the waterbody domain. This resolution is a compromise to ensure the accuracy of results and a suitable run time. The depth values are assigned to the grid cells by using the formerly prepared depth raster layer (Fig. 1) generated from a contour map given by^38^. An interpolation is made to fill-up any empty cell on the grid. To reduce the time and effort of calculations, dry cells which represent little islands and/or dry areas that exist into the waterbody’s domain are blocked.

Simulation of a whole year starts on the 1st of February 2013 (01 02 2013 00:00) and ends on the 31st of January 2014 (31 01 2014 23:00). The time frame is determined based on available data^36–38^. Forces working on the waterbody are defined to be salinity, temperature, and wind. The salinity of the two basins (the Upper and the Lower Lakes) are completely different. The Upper Lake has an average salinity value of 1.6‰ while that of the Lower Lake is 15.6‰ according to the collected field data from monitoring stations provided by the Egyptian Lakes’ Monitoring Program^36^. This detail is well represented by defining the salinity values in the form of spatially varied map instead of a solid value in the initial conditions in the beginning of the simulation. Water temperature and water level are also being given in the form of spatially varied maps as there are differences between the two basins of the waterbody. The mean water temperature of the waterbody is about 15.4 °C. The water level is given a value in reference to the mean sea level to reflect the difference in the two basins’ levels (the Upper Lake is at 10 m below the mean sea level while the Lower Lake is at 32 m below the mean sea level).

The roughness Chezy value is assigned to 40 and the eddy viscosity and diffusivity to 0.5 m^2^/s and all can further be adjusted in the calibration step if needed. The heat flux model uses relative humidity (%), air temperature (°C) and total radiation (J/m^2^/s), which is equivalent to (W/s), as the mean forces to determine the heat transfer and calculate the volume loss due to evaporation. Data used in the heat flux model is of high resolution (hourly-based data). Wind force is represented by high resolution data of wind speed (m/s) and direction (degrees). The prevailing wind is coming from the north and the average wind speed is about 4.4 m/s (Table 3).

**Table 3:** Assumptions, boundary conditions and initial conditions for the model set-up,

Parameter	Value
Model dimensionality	2D
Grid	
Cell size	75 × 107
Points in M-direction	188
Points in N-direction	200
Bathymetry	0.2–24.5 m
Time frame	
Simulation time	1 year
Start time	01 02 2013 00:00
End time	31 01 2014 23:00
Time step	0.3 min
Processes	Temperature, salinity and wind
Open boundaries	None
Initial conditions	
Temperature	15.4 °C
Salinity	
Upper Lake	1.6‰
Lower Lake	15.6‰
Water level	
Upper Lake	− 10 m
Lower Lake	− 32 m
Physical conditions	
Gravity	9.81 m^2^/s
Water density	999.07 kg/m^3^
Air density	1 kg/m^3^
Wind drag coefficient	0.0025 m/s
Chezy roughness	40 m^2^/s
Eddy viscosity	0.5 m^2^/s
Eddy diffusivity	0.5 m^2^/s
Heat flux model	Absolute flux, total solar radiation
Discharges	
Main discharge	5 m^3^/s
Pump station	1.76 m^3^/s
Discharge to the fish farms	0.26 m^3^/s

In the set-up process, three discharge sources are determined and their data are obtained from^30^. These sources are:

- The main discharge (5 m^3^/s on average).
- The pump station (1.76 m^3^/s on average).
- The discharge to the fish farms (0.26 m^3^/s on average).

As the area is hyper-arid, rainfall amounts are ignored in the simulation process as a source of freshwater input.

Many observation points are assigned to the grid to get a time series data throughout the simulation about the different aspects of the waterbody. The location of the observation points is the same as that of the monitoring stations for the Egyptian Lakes’ Monitoring Program^36^ to facilitate comparison. The simulation output data is selected to be on a daily basis to reduce the analysis time and effort and the space of storage as well.

#### Model calibration

The model output values are compared to reference values to decide whether the model is working properly or needs modifications. The main item used for comparison is the water level as it is the main indicator for the hydrodynamics of the waterbody and when simulated properly, it is easier to adjust other parameters. The simulated values of the water level are compared with the values given in literature^25^ who used two sets of data, one that is given by the hydraulic Research Institute (HRI), Ministry of Water Resources and Irrigation^53^ and the other is developed using STELLA modelling environment. The temperature and salinity values are compared to the monthly-based values given in EEAA reports^36,37^. The correlation between the measured and the simulated values for water level, temperature and salinity is evaluated using two error matrices: Root Mean Square Error (RMSE) (Eq. 4) and Average Mean Error (AME) (Eq. 5). Acceptable errors were adopted from previous studies of similar hydrodynamic models of lakes of the same regions^18,25,30,47,54–63^. In addition, two previous models were compared to the results of this study^25^.

4 $$RMSE=\sqrt{\sum_{i, j=1}^{n}{\left({X}_{{m}_{i}}-{X}_{{s}_{j}}\right)}^{2}},$$

where, ${X}_{{m}_{i}}$ is the measured value of water level, temperature or salinity number i, ${X}_{{s}_{j}}$ is the simulated value of water level, temperature or salinity number j and n is the number of readings used in calculating the RMSE matrix.

5 $$AME=\sum_{i, j=1}^{n}ABS ({X}_{{m}_{i}}-{X}_{{s}_{j}})/n,$$

where, ${X}_{{m}_{i}}$ is the measured value of water level, temperature or salinity number i, ${X}_{{s}_{j}}$ is the simulated value of water level, temperature or salinity number j and n is the number of readings used in calculating the RMSE matrix.

After setting-up the model and calculating the error matrices, a decision about the needed amendments is made to adjust the water level in a process known as calibration.

Model calibration is performed by using water level ground truth values^25^ to evaluate the model output using RMSE and AME error matrices. Alteration in values of roughness and viscosity is made, one factor at a time, to attain the minimum error possible. If the system is not sensitive to roughness and/or viscosity alteration, another factor can be checked such as the discharges for example.

#### Model validation

To validate the model, its performance is tested using a set of data that is different from that used for the model set-up and calibration and error matrices are calculated. The new set of data includes meteorological data for 2014–2015 and distribution maps for temperature and salinity at the beginning of the simulation as initial conditions. All other parameters are kept the same as the calibrated model. Once the model validation is acceptable, it can be used for prediction.

### Prediction of Wadi El-Rayan Lakes by the year 2050 under RCP 2.6 and RCP 8.5 scenarios

Upon validating the model, it was employed for predicting the climate change impacts on the hydrodynamic characteristics of the lakes by modifying the present meteorological data by global projected climate change data^40^ according to two RCP scenarios (RCPs 2.6 and 8.5) under pre-determined set of assumptions. RCP 2.6 and RCP 8.5 are two Representative Concentration Pathways (RCPs) that depict possible future greenhouse gas (GHG) emissions scenarios and their impacts on global temperatures, climate patterns, and ecosystems. While RCP 2.6 represents the most ambitious mitigation scenario, RCP 8.5 represents the most pessimistic scenarios with high-emissions. The assumptions for the prediction of climate change impacts on the lakes are:

- The surface area of the lake is the same in the calibrated model.
- The initial conditions for the prediction model are the conditions in the end of the simulation of the calibrated model.
- No more agricultural lands are added to those depending on the lake water for irrigation.
- No more aquaculture added to those depending on the lake water.
- The inflow to and the intake from the lake are the same as those of the calibrated model.

Determining these assumptions are to make sure that no factors other than the climatic factors affect the waterbody in the prediction process and all changes that the waterbody gets through in this process is merely due to climate change.

The time from 2020 to 2050 is rather long period of time. Therefore, in simulation this time is divided into three stages each of which is 10-years span. A simulation of one year long containing the average climatic data for these 10 years is used to represent one stage. Meaning that the first stage starts from 2021 to 2030, for instance, is represented by a whole year run containing the average climatic data for these years after excluding the outliers of course. The output of water level, temperature and salinity for each stage is used as the initial conditions for the next one. This prediction process is made twice, one for the RCP 2.6 scenario and the other for the RCP 8.5.

The global projected climate change data^40^ are monthly-based parameters from January 2020 till December 2050. These data have been processed and prepared for further use. Climate change data include air temperature (°K), which transformed to Celsius degree (°C) values, relative humidity (%), surface downwelling clear sky shortwave radiation (W/m^2^) and wind stress (Pa).

Wind speed is transformed from wind stress using the relationship that combine them both which is:

6 $${\tau }_{(N/{m}^{2})} ={{\rho }_{a}}_{(Kg/{m}^{3})}{C}_{D}{U}_{{}_{(m/s)}}^{2},$$

where $\tau$ is the wind stress in $\text{N}/{\text{m}}^{2}$ ($1 \text{N}/{\text{m}}^{2}=1 \text{Pascal} (\text{Pa})$), ${\rho }_{a}$ is the air density in $\text{kg}/{\text{m}}^{3}$, ${C}_{D}$ is the non-dimensional drag coefficient and $U$ is the wind speed at 10 m above the water surface in $\text{m}/\text{s}$. Air density ${\rho }_{a}$ values range from 1.2 to 1.3 kg/m^3^ according to temperature, pressure and humidity^64^. Wind stress drag coefficient C_D_ value is directly related to wind speed. When wind speed increases, the C_D_ value increases as well. Its value ranges from 1 × 10^–3^ to 1.5 × 10^–3^ in case of wind speed up to 15 m/s^64–68^. The C_D_ value used in this work is the global value of 1.25 × 10^–3^^67^.

After modeling the CO_2_ solubility at different temperatures and salinities, the model is used to generate seasonal surface CO_2_ solubility maps for the current and future situations. The seasonal temperature and salinity maps used to generate these solubility maps are obtained from the successful Delft3d-FLOW hydrodynamic model which is calibrated, validated and used for prediction as described in the former two sections. Water depth, temperature and salinity maps generated from validation and prediction processes are used as inputs to this work (Table 2).

### Estimation of change in the Wadi El-Rayan Lakes’ CO_2_ sink capacity

To estimate the amount of change in the Wadi El-Rayan Lakes CO_2_ sink capacity, there is a need to estimate the lakes’ current and future potentials to dissolve CO_2_. To quantitative estimate the soluble CO_2_ in the lakes, four assumptions are made for simplification purposes:

1. Temperature, salinity and water depth for a month in a fixed point of time is used to represent this month. In our case, the Delft3D-FLOW output raster maps, in a fixed time step (15th of the month, 01:00:00 or 00:00:00) are taken to represent this month. Four months are selected (Feb, May, Aug and Nov) to represent the four seasons of the year.
2. The waterbody is divided into water columns, each of which has a surface area of 30 m × 30 m (raster cell dimensions) and a depth (raster Z value) that differs according to the cell spatial location (x and y coordinates of the surface cell).
3. The temperature and salinity distribution all along the water column of one cell is homogenous and its value is the value of the surface cell.
4. The CO_2_ solubility is following the fit equation generated in “Data and methodology” under its limiting conditions of the amount of CO_2_ contained in the moist air and the air pressure.

The amount of CO_2_ soluble in a water column is a function of its solubility (mg/L) for a specific surface cell (in the solubility raster maps) and the water volume (L) of the water column of this cell (the Z value of the depth raster maps). The total soluble CO_2_ of the waterbody is the sum of soluble CO_2_ in all water columns of the image. After calculating the soluble CO_2_ in the lakes for the current and future conditions, a two-level analysis is made to estimate the changes in Wadi El-Rayan sink capacity according to the water column capacity and the total waterbody capacity. As the data for this work is spatial data, ArcGIS 10.8 is used for their processing and analysis. Statistical analysis is performed using OriginLab and MS-Excel.

- Water Column Capacity Level uses the mean water column capacity to dissolve CO_2_ from the atmosphere at different circumstances which are season, location and time. Data of water columns are distributed normally depending on their mean and standard deviation to compare their attitude under the three specified circumstances as follows:
  o Season to estimate which season [represented by the four months of Feb, May, Aug and Nov] has the maximum capacity to dissolve the CO_2_ from the atmosphere, regardless of the time [year 2014, 2050 (RCP 2.6) and 2050 (RCP 8.5)] and location [Upper Lake and Lower Lake]. Data of one season (represented by one month) in all times and locations are considered as one set of data and their mean and standard deviation are calculated. Four normal distribution graphs are made representing Feb (winter), May (spring), Aug (summer) and Nov (fall).
  o Location to estimate which lake has the maximum capacity to dissolve the CO_2_ from the atmosphere, regardless of the time [year 2014, 2050 (RCP 2.6) and 2050 (RCP 8.5)] and season [Feb, May, Aug and Nov]. All data of the Upper Lake are considered as one set of data in all times and seasons. The same is made for the Lower Lake. The mean and standard deviation are calculated for each lake and the data are distributed normally to bring two graphs representing the Upper Lake and the Lower Lake.
  o Time to estimate which of the studied years [year 2014, 2050 (RCP 2.6) and 2050 (RCP 8.5)] has the maximum capacity to dissolve the CO_2_ from the atmosphere, regardless of the location [Upper Lake and Lower Lake] and season [Feb, May, Aug and Nov]. Therefore, all data of one year are considered as one set of data, their mean and standard deviation are calculated and data are distributed normally to bring three graphs representing year 2014, year 2050 (according to RCP 2.6) and year 2050 (according to RCP 8.5).
- Total Capacity Level to estimate how much loss in the CO_2_ sink of Wadi El-Rayan Lakes is due to climate change. This is attained by computing the sum of all water column capacities for each lake in the current (year 2014) and future (2050 according to both scenarios RCP 2.6 and 8.5) conditions.

## Results and discussion

### Model for CO_2_ solubility in water

**Fig. 3:**
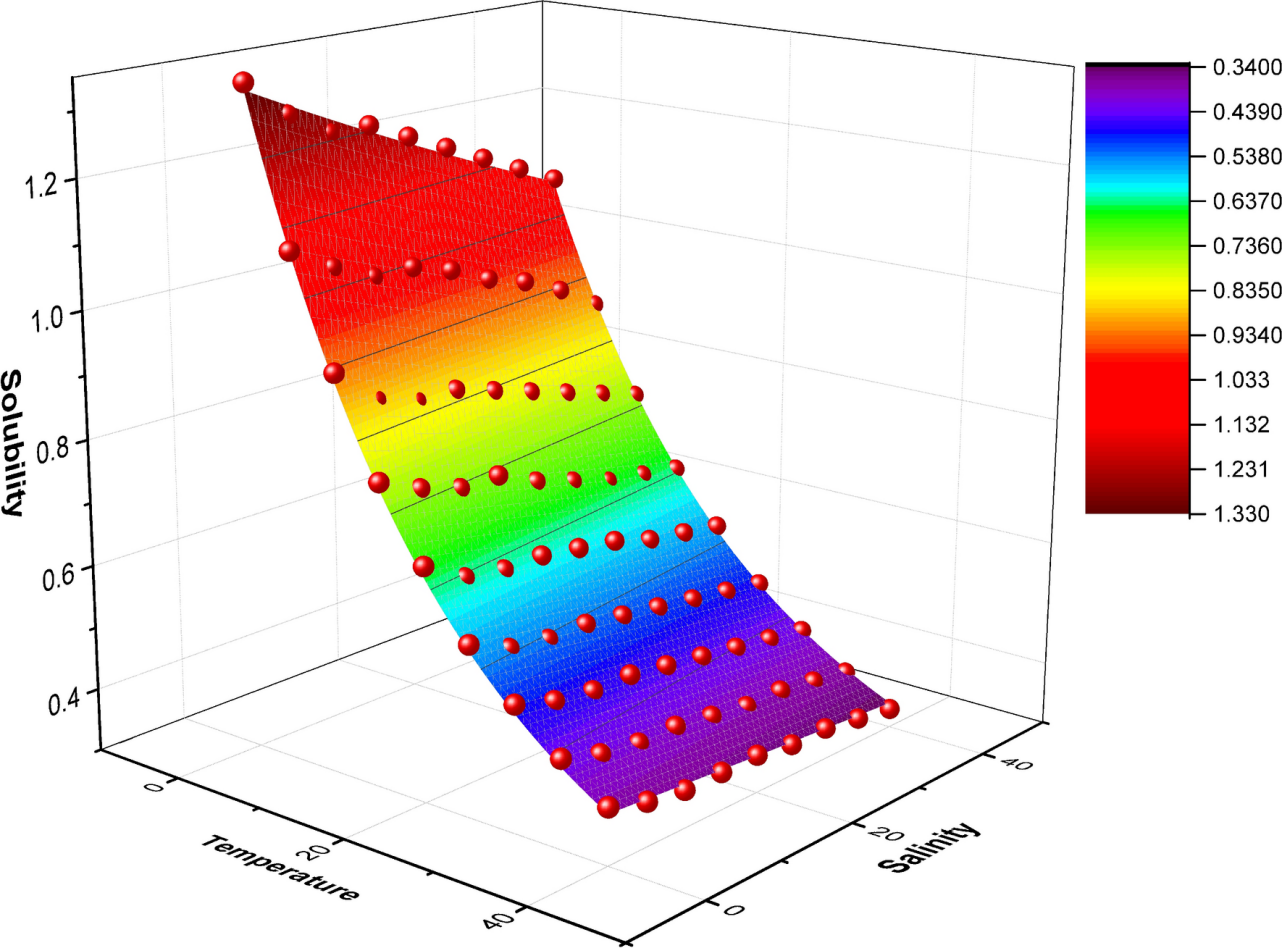
Surface fit for CO_2_ solubility (mg/L) as a function of temperature (°C) and salinity (‰).

The CO_2_ solubility (mg/L), as a function of both temperature (°C) and salinity (‰), has been modelled using non-linear surface fit (Fig. 3) of a general formula as (Eq. 7) using the OriginLab statistical analysis software. Eighty-one points are used for the model fit representing different combinations of temperature and salinity conditions with 9 iterations to reach the fitting. The surface fit curve is found to be represented by the equation (Eq. 8) with high correlation (R^2^ = 0.99886), low residual sum of squares (RSS = 0.00669) and Chi-square tolerance value of 10^–9^ is reached.

7 $$Z= {Z}_{0}+B {e}^{(- \frac{X}{C} - \frac{Y}{D})}$$

where Z_0_, B, C and D are the constants of fitting of the model while X, Y and Z are the variables.

8 $$C=0.19605+1.13251 {e}^{(- \frac{T}{21.99386} - \frac{S}{165.67292})}$$

where **C** is the Solubility of carbon dioxide (mg/L) in water at different temperatures **T** (°C) and salinities **S** (‰) exposed to moist air containing 0.04% carbon dioxide at a total air pressure of 760 mm Hg. This equation is used later to generate CO_2_-solubility raster images (Figs. 8, 9 and 10) which would be used later with bathymetry maps to estimate soluble CO_2_ in water column and in the waterbody as a whole assuming homogenous temperature and salinity distribution through water columns as identical as surface cells.

### Developed hydrodynamic model

#### Model set-up and calibration

Outputs of water level, temperature and salinity are compared to reference data. Simulated water level values are compared to^25^ in order to evaluate the model. The pattern of the water level fluctuations for both lakes are close to those of the reference curves but the simulated values are about 10 cm higher than the actual ones for the Upper Lake and about 70 cm less than the actual values in case of the Lower Lake. Therefore, model still needs adjustments to bring the water level closer to the reference especially for the Lower Lake. The error matrices for the Upper Lake water level are 0.18 and 0.2 while those of the Lower Lake are 0.3 and 0.47 for the RMSE and the AME, respectively, which are all needed to be further reduced in the calibration process.

**Fig. 4:**
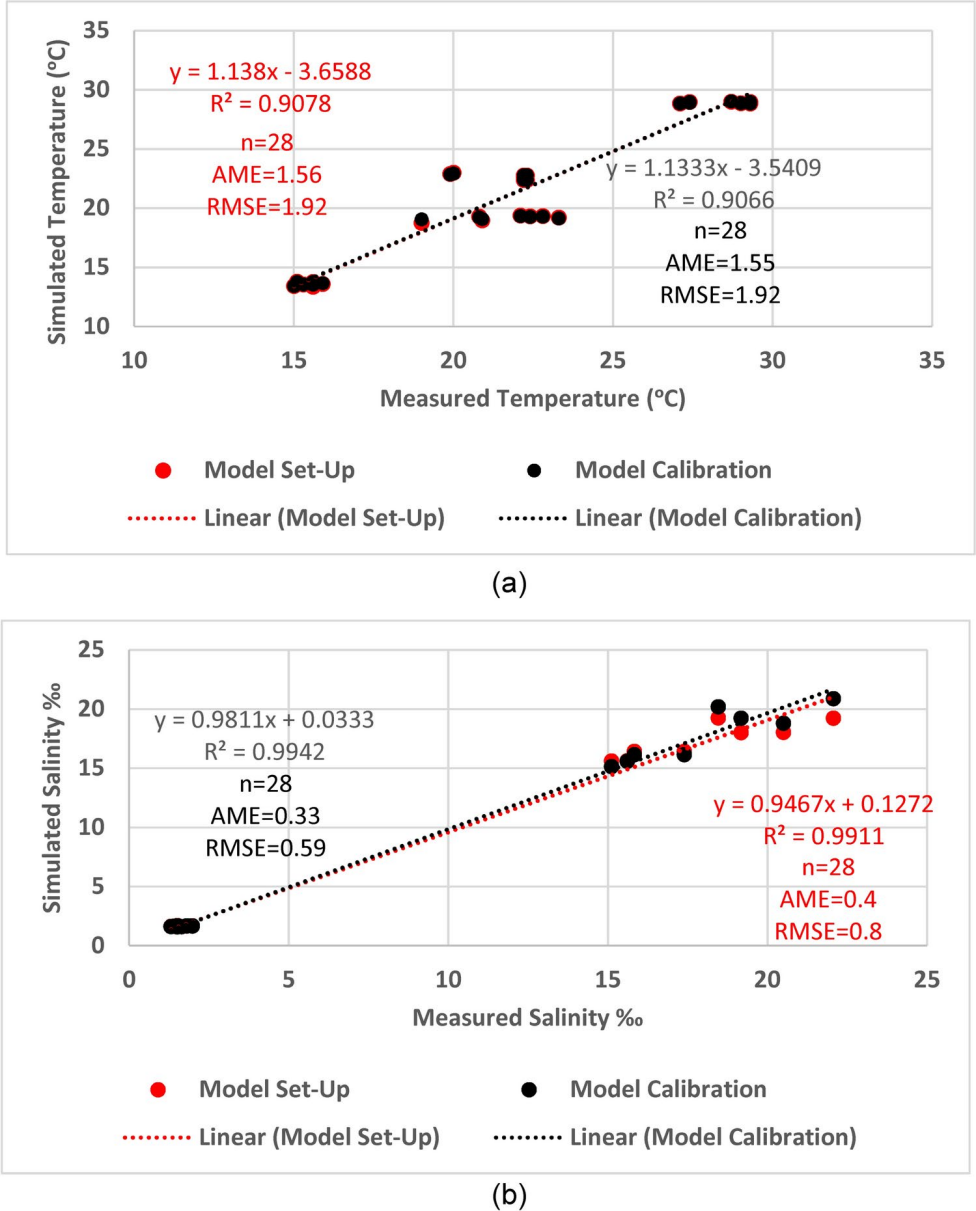
Correlation between measured and simulated (**a**) temperature (°C) and (**b**) salinity (‰) in the model setup and calibration processes.

Both simulated temperature and salinity values are compared to the monthly-based values given in EEAA reports^36,37^. High correlation is found between the simulated and the measured values for both temperature (R^2^ = 0.91, n = 28 AME = 1.56, RMSE = 1.92) and salinity (R^2^-0.99, n = 28 AME = 0.4, RMSE = 0.8) with acceptable error as determined by two error functions which are the average mean error (AME) and the root mean square error (RMSE) (Fig. 4).

To calibrate the model, modifications have been made to the values of roughness and viscosity, one alteration at a time, and the results of water level showed that the model is not sensitive to both of these parameters. By reviewing the discharges of the waterbody, there have been some minor water sources that have been neglected in the model set-up process as there are no available data for them. For example, literature refers to withdrawal of some of the Upper Lake’s water to irrigate reclaimed lands to the east^29^. The location and flow data of this minor drain is unavailable. Also, there is several minor water connections from a nearby fish farm to the north east of the Lower Lake that likely to disperse its surplus water. Likewise, the excess irrigation water of the nearby reclaimed lands drains into the Lower Lake following the landscape gradient^28^. Again, there are no available data about these water quantities that can be reached which would cause some error in water level estimation. To compensate for that estimation error, inflow and outflow has been changed by a fixed percentage (10%, 20% …) and the output of the model is tested against the ground truth data of water level. It is a series of trial-and-error experiments for the model to attain calibration. Each time, one parameter is changed and other parameters are kept the same then run the model and test the result. Calibration has been reached under these conditions:

- Reduce the inflow to the Upper Lake by 10%.
- Increase the intake by the pump station and fish farms by 10%.
- Introduce a drain in the north east of the Lower Lake (LL) to add the water taken from the Upper Lake plus a constant value of 0.2 m^3^/s.

**Fig. 5:**
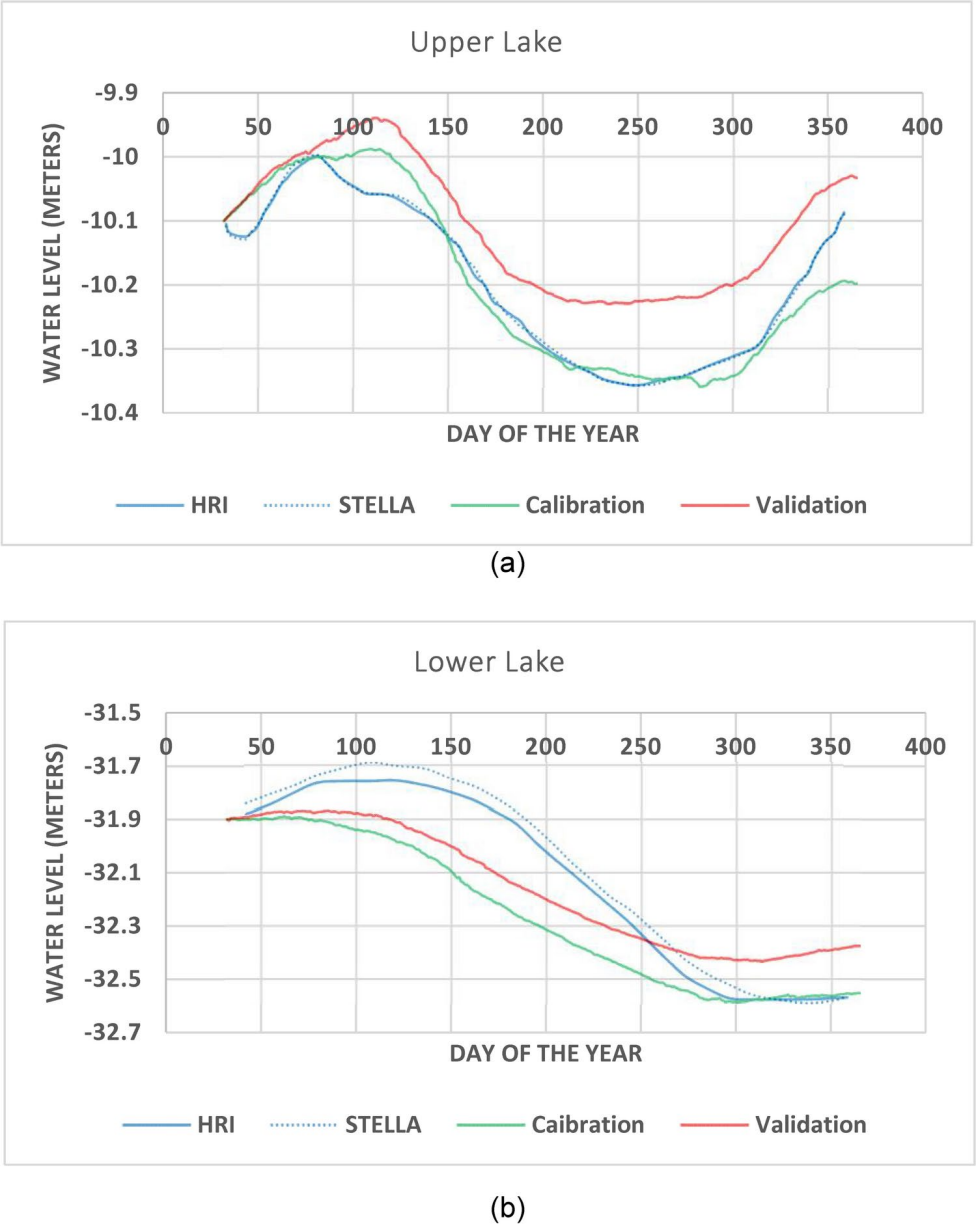
Water level of Wadi El-Rayan Lakes: (**a**) the Upper Lake and (**b**) the Lower Lake in the model calibration and validation processes compared to reference curves.

After adjusting the water inflow and outflow, calibrated model shows a convenient accord between the simulated water level values and the reference values with little error for the Upper Lake (AME = 0.03, RMSE = 0.19) and the Lower Lake (AME = 0.04, RMSE = 0.23) (Fig. 5). Data of monthly averaged salinity and temperature given by the EEAA are used to calibrate the salinity and temperature of the model. There is high correlation between the measured and simulated temperature (R^2^ = 0.91, n = 28, AME = 1.55, RMSE = 1.92) and salinity values (R^2^ = 0.99, n = 28, AME = 0.33, RMSE = 0.59), for the calibration process (Fig. 4) with acceptable error of simulation for both**.**

#### Model validation

**Fig. 6:**
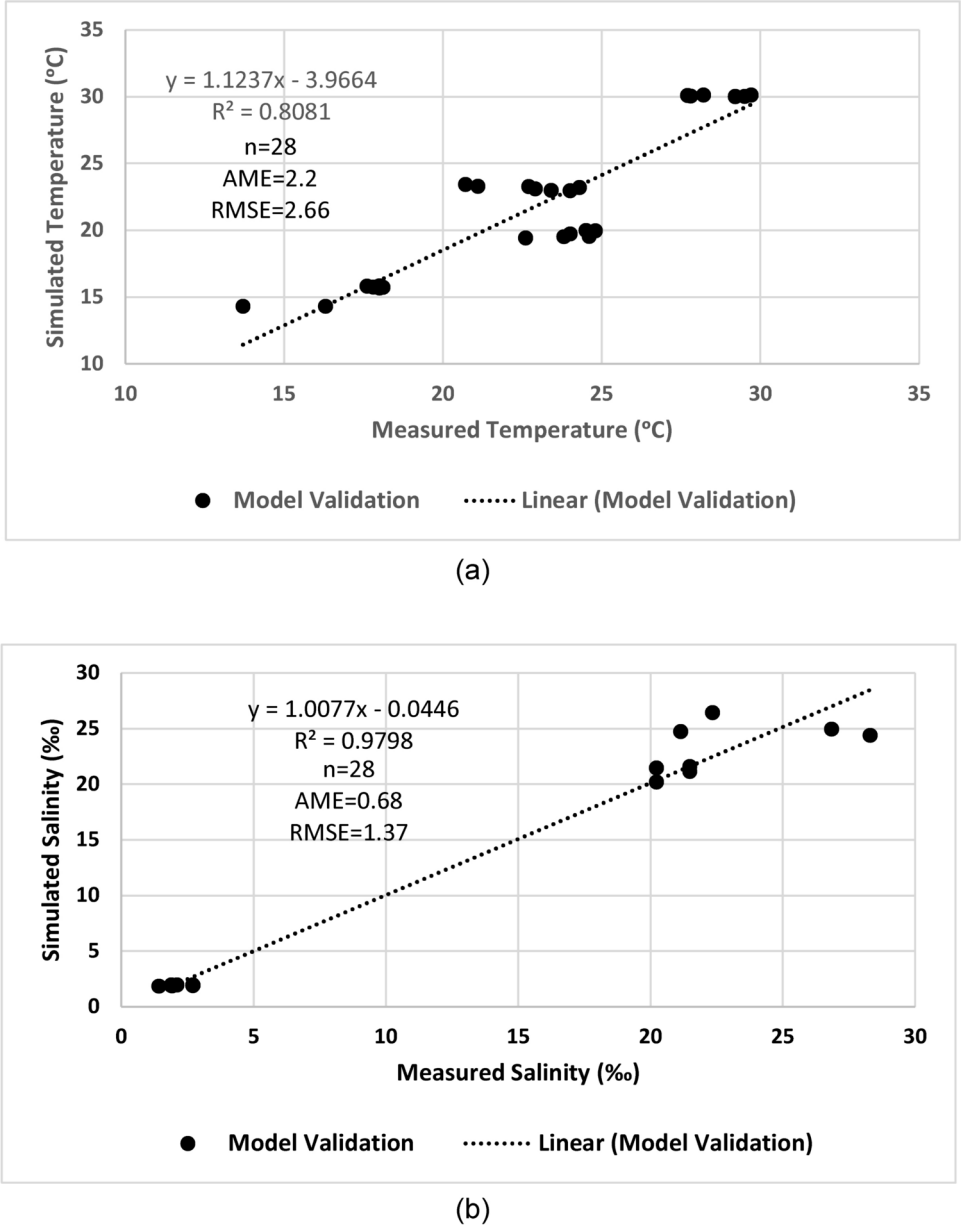
Correlation between measured and simulated (**a**) temperature (°C) and (**b**) salinity (‰) in the validation process.

The calibrated model is used to simulate another set of meteorological data (2014–2015). The salinity and temperature of the initial conditions are adjusted to those of this period of time. All other factors are kept the same as the calibrated model. Regarding the water level, the AMEs are 0.08 and 0.16 and the RMSEs are 0.9 and 0.17 for the Upper and the Lower Lakes, respectively, which are considered accurate (Fig. 6). For temperature and salinity, there is a high correlation between measured and simulated values (R^2^ = 0.81, n = 28, AME = 2.2, RMSE = 2.66 for temperature and R^2^ = 0.98, n = 28, AME = 0.68, RMSE = 1.37 for salinity) (Fig. 6). Regarding the reliability and the availability of data, the model validation is acceptable and can be used for prediction.

### Water depth, temperature and salinity of Wadi El-Rayan Lakes under climate change scenarios

**Fig. 7:**
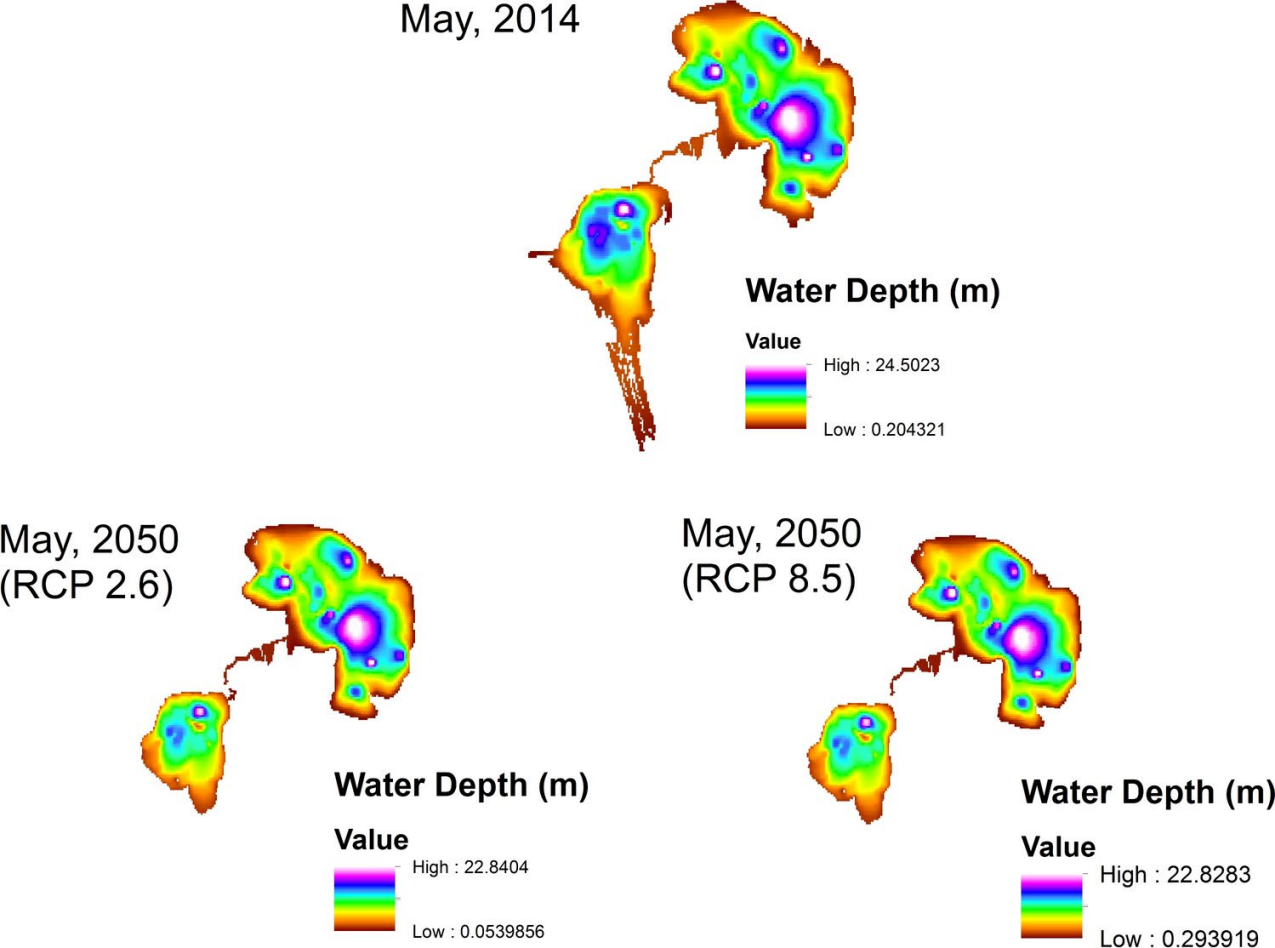
Water depth of Wadi El-Rayan Lakes in 2014 and 2050 (RCPs 2.6 and 8.5).

After introducing the projected meteorological data according to the two RCP scenarios (RCP 2.6 and RCP 8.5). A remarkable decrease in the water depth has been identified (Fig. 7) for both lakes indicating notable water volume loss mainly for the Lower Lake due to evaporation. The loss of water is higher, of course, in case of RCP 8.5 scenario but in general both scenarios, the nethermost and the outmost, lead to close outcomes. This can be related to the nature of the water itself which responds slowly to changes in atmospheric temperature. The absorbed heat by the lakes’ water accelerates the evaporation rates and caused the dryness of shallow parts of the lakes. As a result of increased evaporation, the lack of another source of freshwater entering the lake and also because the lake continues to receive agricultural and fish-farm drainage, the salinity has increased remarkably mainly in the Lower Lake which has the highest salinity from the beginning. The salinity of the Upper Lake is expected to increase by about 1 ppt to reach about 2.5 ppt in value. This cannot be compared to the increase that is expected for the Lower Lake which may reach 60 ppt.

### Estimated soluble CO_2_ in water column

**Fig. 8:**
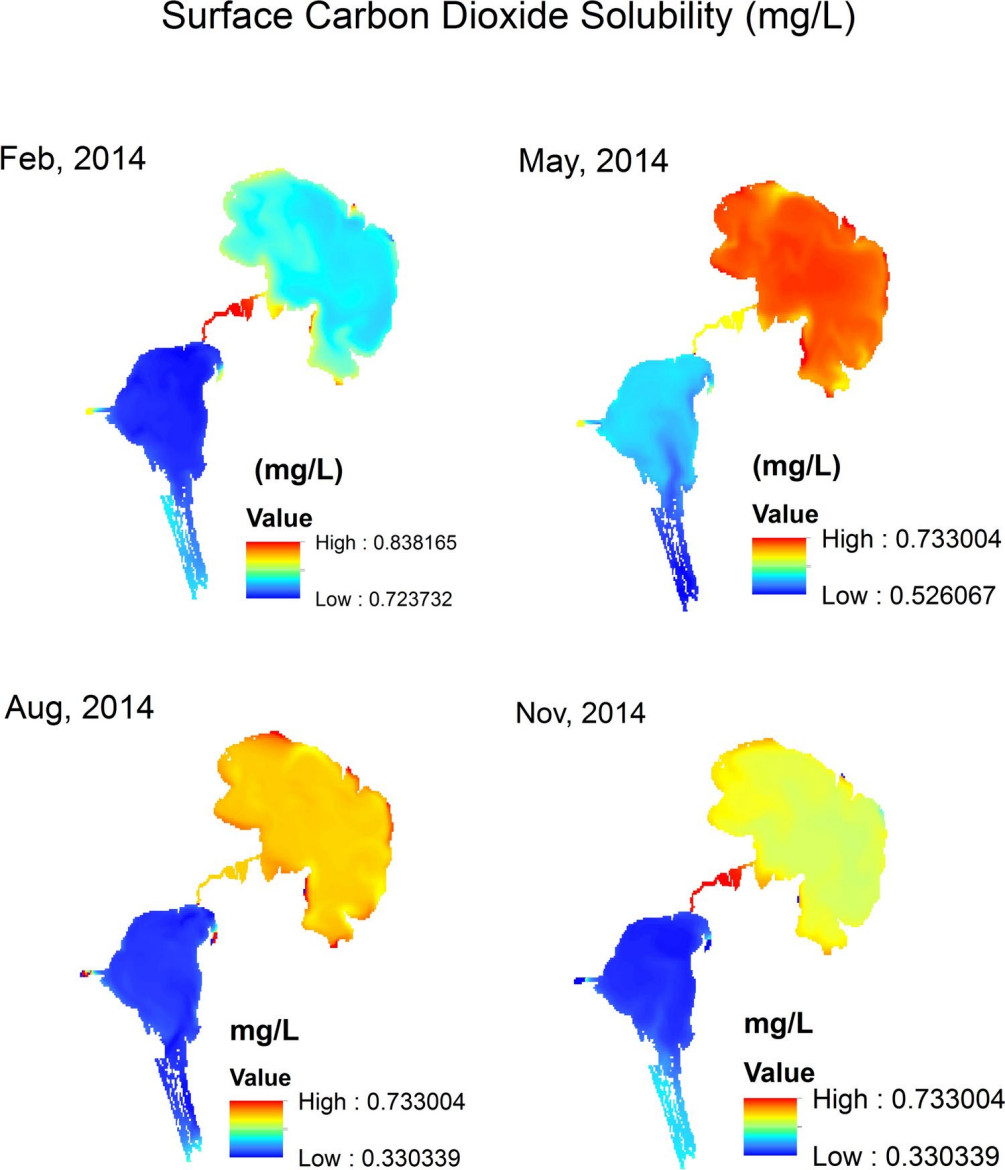
CO_2_ solubility (mg/L) in Wadi El-Rayan Lakes according to given conditions of temperature (°C) and salinity (‰) for the year 2014.

**Fig. 9:**
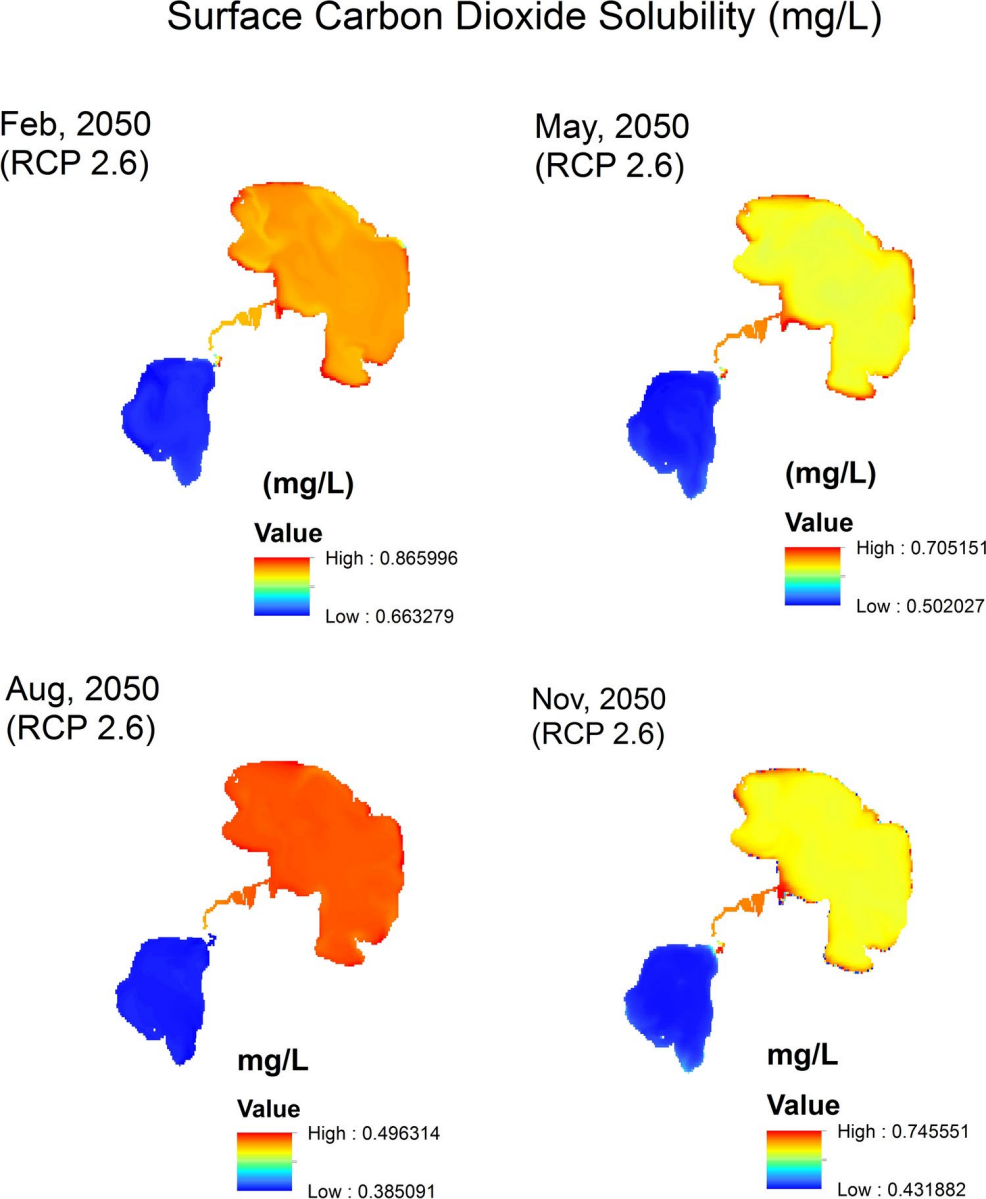
CO_2_ solubility (mg/L) in Wadi El-Rayan Lakes according to predicted conditions of temperature (°C) and salinity (‰) for the year 2050 (RCP 2.6).

**Fig. 10:**
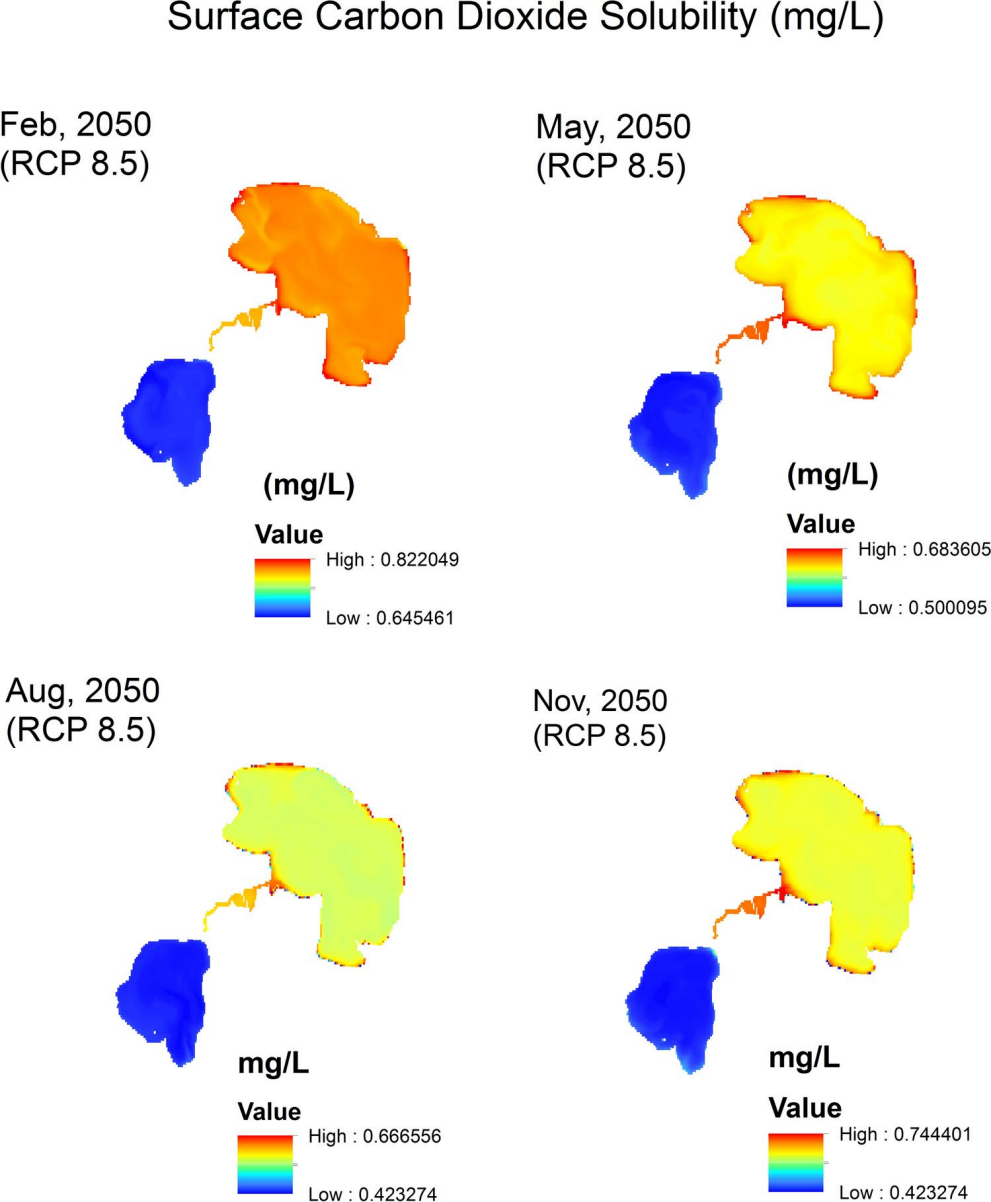
CO_2_ solubility (mg/L) in Wadi El-Rayan Lakes according to predicted conditions of temperature (°C) and salinity (‰) for the year 2050 (RCP 8.5).

**Fig. 11:**
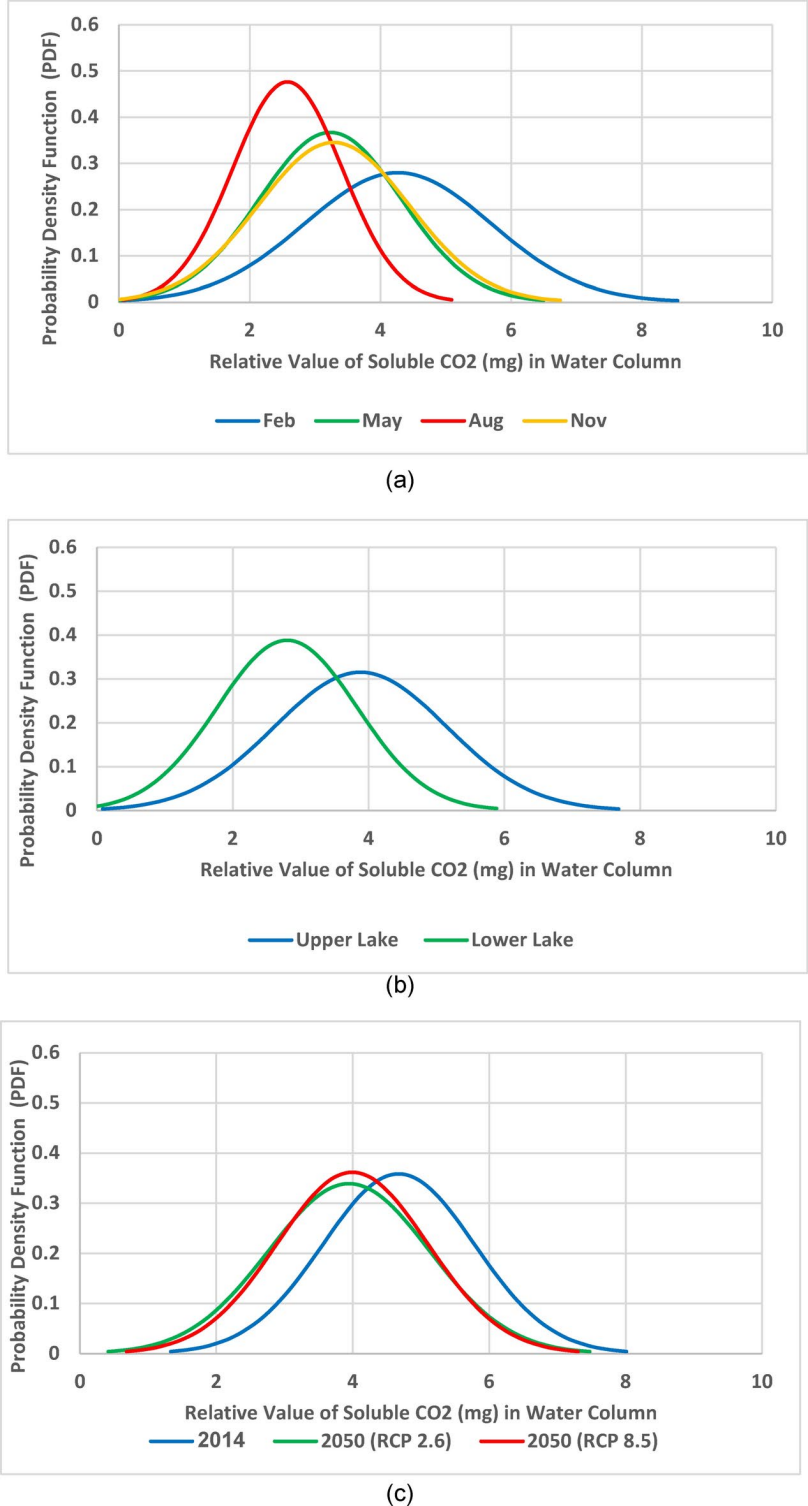
Probability density functions for the soluble CO_2_ (mg) in water column in respect to (**a**) season represented by the four months of Feb, May, Aug and Nov, (**b**) location represented by the Upper and the Lower Lakes and (**c**) time represented by the years 2014, 2050 (RCP 2.6) and 2050 (RCP 8.5).

Surface CO_2_ solubility (mg/L) raster maps (Figs. 8, 9, 10) are generated by applying the mathematical model (Eq. 8) to current and future conditions. In these solubility maps, each pixel represents the solubility of CO_2_ in the given water surface temperature and salinity for this pixel of dimensions 30 m × 30 m. Homogenous distribution of temperature and salinity is assumed throughout the water column. Therefore, the soluble CO_2_ in the water column of 30 m × 30 m surface area is estimated by calculating the water volume (L) of the water column using the water depth raster maps and multiplying the surface area by the water depth for each pixel to generate the soluble CO_2_ maps. Data extracted from the different soluble CO_2_ maps are summarized in Table 4. These data are statistically analyzed using the normal distribution by fixing two parameters and analyze the change in the third one. The three parameters are: (a) *season* represented by the four months of Feb, May, Aug and Nov (Fig. 11a), (b) *location* represented by the Upper Lake and the Lower one (Fig. 11b) and finally (c) *time* represented by the years 2014, 2050 (RCP 2.6) and 2050 (RCP 8.5) (Fig. 11c).

**Table 4:** Seasonal soluble CO_2_ in water column (mg) for Wadi El-Rayan’s Upper and Lower Lakes in 2014 and 2050 according to RCP 2.6 and RCP 8.5.

Lake	Month	2014		2050 (RCP 2.6)		2050 (RCP 8.5)	
		Soluble CO_2_ in water column (mg)		Soluble CO_2_ in water column (mg)		Soluble CO_2_ in water column (mg)	
		Mean	SD*	Mean	SD*	Mean	SD*
Upper Lake	Feb	6.49	3.53	6.06	3.67	5.97	3.54
	May	5.15	2.81	4.47	2.74	4.50	2.70
	Aug	4.07	2.26	3.41	2.19	3.74	2.36
	Nov	5.52	3.04	4.58	2.89	4.60	2.86
Lower Lake	Feb	5.23	3.30	4.47	2.32	4.39	2.23
	May	3.90	2.50	3.29	1.75	3.29	1.71
	Aug	3.04	2.02	2.38	1.40	2.57	1.48
	Nov	3.97	2.68	2.90	1.76	2.88	1.74

*SD Standard deviation of the image data.

Figure 11a indicates that the month of February, representing the winter season, has the maximum capacity to absorb CO_2_ from the atmosphere regardless of the lake and/or the year of simulation while the month of August has the minimum capacity. This is mainly because of the low water temperature during the winter season. As temperature gets lower, the CO_2_ ability to dissolve in water increases and vice versa. Figure 11b reveals that the Upper Lake is always a better sink for CO_2_ than the Lower one. The core factor in this case is not the temperature but the salinity as both lakes’ temperature variations are close. The Upper Lake salinity is much lower than that of the Lower one. The lower the salinity is, the higher the CO_2_ solubility would be. Figure 11c denotes that there is a retreat in the lakes’ capacity to dissolve CO_2_ from the atmosphere in 2050 according to both scenarios RCP 2.5 and 8.5 when compared to their capacity in 2014. The is essentially related to the increase in water temperature as a result of the increased air temperature due to climate change.

### Potential changes in the CO_2_ sink capacity due to climate change

**Fig. 12:**
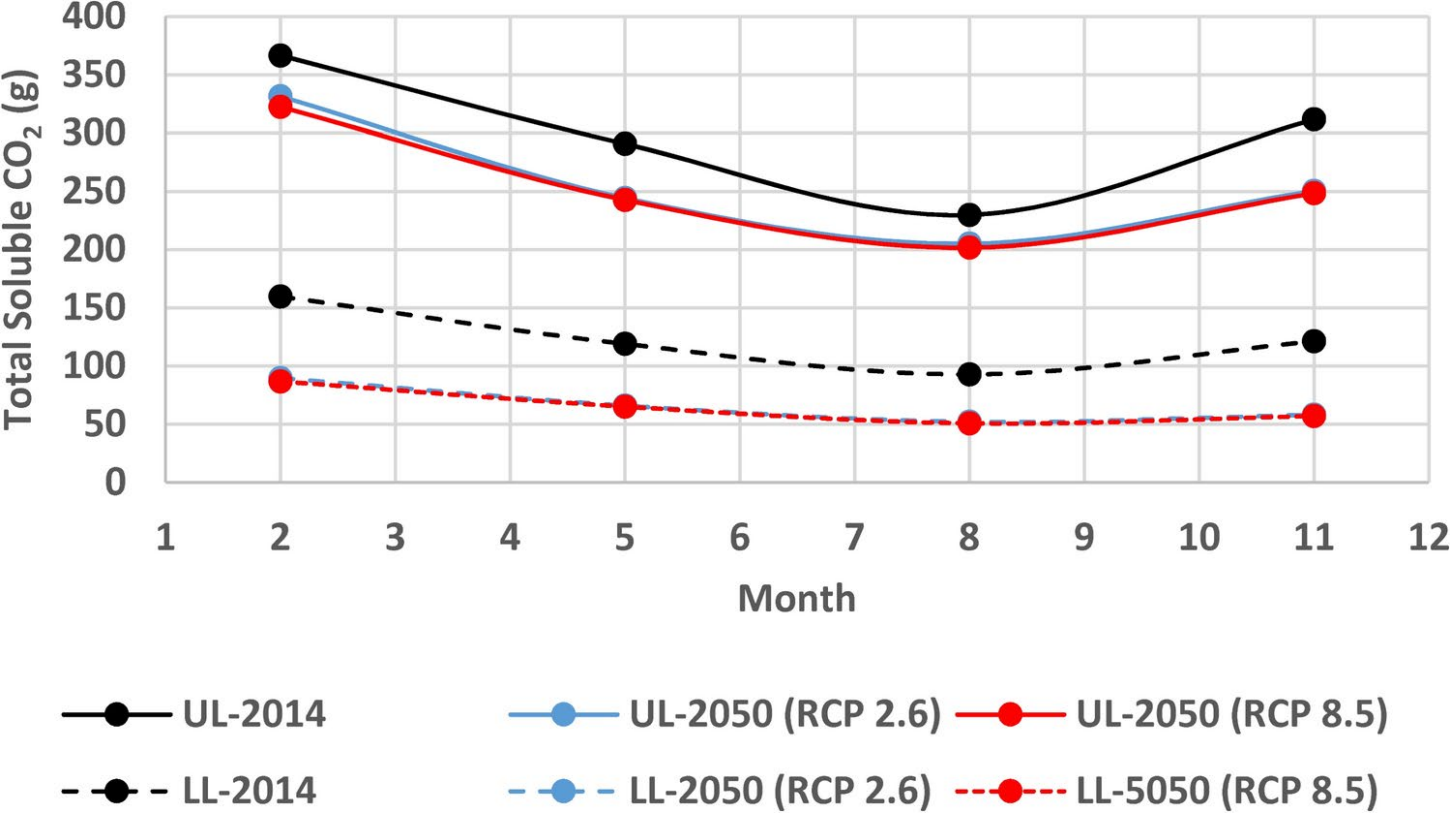
Carbon dioxide sink capacity of the Upper Lake (UL) and Lower Lake (LL) (the amount of soluble CO_2_ (g) in these lakes) for the years 2014, 2050 (RCP 2.6) and 2050 (RCP 8.5).

To get the total soluble CO_2_ in the lakes, summation of the amount of soluble CO_2_ in all water columns is attained for each image which represents the average soluble CO_2_ in this month (Table 5 and Fig. 12).

**Table 5:** CO_2_ Sink capacity of Wadi El-Rayan Lakes in current and future conditions and the percentage of change in their capacities due to climate change.

Month	Sink capacity represented by the amount of soluble CO_2_ (g) in water						% Change in Sink’s capacity from 2014 to 2050 (RCP 2.6)		% Change in Sink’s capacity from 2014 to 2050 (RCP 8.5)	
	2014		2050 (RCP 2.6)		2050 (RCP 8.5)					
	Upper Lake (UL)	Lower Lake (LL)	Upper Lake (UL)	Lower Lake (LL)	Upper Lake (UL)	Lower Lake (LL)	Upper Lake (UL)	Lower Lake (LL)	Upper Lake (UL)	Lower Lake (LL)
2	366	160	331	90	322	87	− 10%	− 44%	− 12%	− 46%
5	291	119	244	66	243	65	− 16%	− 45%	− 17%	− 45%
8	230	93	205	52	202	51	− 11%	− 44%	− 12%	− 45%
11	312	121	250	58	248	57	− 20%	− 52%	− 20%	− 53%
Average	300	123	258	66	254	65	− 14%	− 46%	− 15%	− 47%

According to results, Both Wadi El-Rayan Lakes have the ability to dissolve about 400 g in average of atmospheric CO_2_ monthly and the Upper Lake capacity to dissolve CO_2_ from the atmosphere is twice that of the Lower Lake in 2014 and four times higher than it in 2050 under RCP2.6 and RCP8.5 Scenarios. This is mainly due to the difference in salinities (‰) between the two lakes. The higher the salinity is, the lower the CO_2_ solubility in water would be. The lakes perform better, as CO_2_ sinks, in the cold seasons than the hot ones. This is because of the fact that the lower the temperature is, the higher solubility would be. The CO_2_ sink of Wadi El-Rayan Lakes is expected to lose more than 23% of its functions by the year 2050 according to RCP 2.6 and 25% according to RCP 8.5 and most of the loss is from the Lower Lake. Meaning that its ability to dissolve CO_2_ from the atmosphere would decrease by about 23–25% which would affect the climate in Wadi El-Rayan area. The undissolved CO_2_ in lakes’ water, which is a result of changes happened to the lakes due to climate change, would remain in the atmosphere leading to increased greenhouse effect which in turn would lead to an increase in Wadi-El-Rayan Lakes surface water temperature due to the heat exchange in the water-atmosphere interface. Expected increase in water surface temperature leads to more decrease in CO_2_ solubility in water and hence a more decline in the functionality of the CO_2_ sink of Wadi El-Rayan Lakes.

### Potential limitations

Although the proposed integrated GIS and hydrodynamic modelling approach is innovative, easy-to-use and time and cost effective, some limitations may reduce the reliability of the model results. These limitations are associated with the model assumptions and can be summarized in the following points:

- Solubility of CO_2_ in water at different temperatures and salinities are modelled where the waterbody is exposed to moist air containing 0.04% CO_2_ at a total air pressure of 760 mmHg. Any change of the CO_2_ concentration in air and/or the increase or decrease in atmospheric pressure can be a source of errors in the model.
- Homogenous temperature and salinity distribution in water columns of a surface area of 30 × 30 m is assumed. Therefore, thermal stratification of waterbodies can generate estimation errors.
- Biogeochemical interactions are overlooked for simplification purposes which may lead to inaccurate solubility predictions in areas with high biological activities.
- Hydrodynamic models are sensitive to the initial and boundary conditions set for temperature, salinity, meteorological data and inflow/outflow rates. Incorrect assumptions and/or data supply in these inputs can propagate errors through the model, especially in long-term prediction.
- It’s worth mentioning that the results of hydrodynamic model for CO₂ solubility under climate change scenarios should be carefully interpreted particularly with the uncertainty associated with climate change scenarios and the long-time span of the prediction

## Conclusion

Hydrodynamic model is essential to understand how a natural water system works. Climate forces (such as wind), inflow and outflow fluxes and density gradient are the driving forces for water movement (hydrodynamics). Properly calibrated and validated hydrodynamic model is a powerful tool to simulate the changes in the parameters of a given waterbody under different climate change scenarios (RCPs 2.6 and 8.5). Data generated by the model such as water depth, temperature and salinity are further analyzed to estimate the ability of the waterbody to dissolve atmospheric CO_2_.

The integrated approach proposed in this paper is a simplified and innovative method to estimate the CO_2_ sink capacity of a waterbody. It depends on integrating physical knowledge about CO_2_ solubility in water (CO_2_ solubility water model) with the spatial analysis of the outcomes of the hydrodynamic model (water depth, temperature and salinity). Spatial analysis relates spatially distributed temperature and salinity of the lakes to the CO_2_ solubility in water model (an outcome of this work) and water depth data to estimate the soluble CO_2_ in water columns and then the total soluble CO_2_ in the waterbody. This approach is far from generalization in estimation as it takes into account the variation in values of temperature, salinity, water depth and the subsequent soluble CO_2_ in water throughout the waterbody. This approach can be extended to other waterbodies and the data used can get more sophisticated as using 3D hydrodynamic model outcomes and also spatial analysis can be extended to more complicated physical solubility models.

Under climate change RCP 2.6 and 8.5 scenarios, CO_2_ sink capacity of Wadi El-Rayan Lakes are expected to decrease by 23% and 25% by the year 2050. The reduced capacity will be more noticeable in the case of the Lower Lake. Such reduced waterbody sink capacity would lead to increased CO_2_ in the atmosphere and hence increases the greenhouse effect. Greenhouse effect would increase atmospheric temperature which in turn leads to an increase in surface water temperature as a result of heat exchange between water and atmosphere. This is a mutual complex process, as atmospheric temperature increases, surface water temperature increases and dissolved CO_2_ decreases, which in turn causes the atmospheric temperature to increase more and so on in a loop action till the waterbody reaches a temperature that it would dissolve no more CO_2_ instead it would start releasing CO_2_ to the atmosphere. It’s worth to mention that increased water temperature not only leads to decreased dissolved gases such as CO_2_ and oxygen which are both important to aquatic biota but also it disturbs aquatic life, affects fish maturation and metabolism and sometimes causes mortality.

In order to conserve this valuable waterbody as a CO_2_ sink, some measures have to be taken to control evaporation and water inflow which in turn would affect water volume, temperature and salinity as well. To decrease evaporation, shade should be provided to surface water by planting trees on banks and growing free-floating vegetation on water surface. Wide-canopy trees along the lake banks is a good suggestion especially when accompanied with introducing free-floating wide-leaf aquatic plants. Both trees and aquatic plants would cover considerable areas of surface water to reduce evaporation, consume CO_2_ in the photosynthesis process (which itself is an added value to the CO_2_ sink), reduce the surface water temperature in their areas which would, in turn, enhance the water circulation as a result of water density gradient between shaded and unshaded areas. Moreover, vegetation would add aesthetic value to the landscape. Likewise, covering the waterbody surface by floating solar panels would block sunlight leading to water surface temperature decrease which in turn can help in limiting evaporation. In such a case, both water conservation and renewable energy production can be addressed.

The water inflow to Wadi El-Rayan Lakes comes originally from Wadi Drain, which contains agricultural drainage water of Fayoum agricultural lands, through a canal and an underground tunnel to flow in the Upper Lake which fills the Lower one by its surplus water. To conserve lakes’ volume, allowing more water quantity to flow to the Upper Lake should be considered seriously in order to compensate for water loss and reduce the degradation of salinity specially in the Lower Lake.

Management recommendations such as controlled evaporation and increased water inflow aim to conserve water volume, decrease temperature and control salinity from getting increased which in turn would mitigate the decline in the CO_2_ sink capacity of the waterbody. The reason is that as the water gets colder and fresher, the more its ability to dissolve CO_2_ would be. Likewise, the larger the water volume gets, the more atmospheric CO_2_ it would dissolve. Therefore, it is necessary to conserve the water volume, temperature and salinity in order to allow the waterbody to maintain its role as being a CO_2_ sink rather than becoming a CO_2_ source.

## Declarations

### Data availability

The datasets generated during and/or analyzed during the current study are available from the corresponding author on reasonable request.
